# Dual energy CT arthrography in shoulder instability: successful iodine removal with virtual non-contrast images and accurate 3D reformats of the glenoid for assessment of bone loss

**DOI:** 10.1007/s00256-021-03916-3

**Published:** 2021-10-03

**Authors:** Christoph Stern, Magda Marcon, Samy Bouaicha, Karl Wieser, Andrea B. Rosskopf, Reto Sutter

**Affiliations:** 1 Radiology, Balgrist University Hospital, Forchstrasse 340, 8008 Zurich, Switzerland; 2 Faculty of Medicine, University of Zurich, Zurich, Switzerland; 3 Department of Orthopaedic Surgery, Balgrist University Hospital, Forchstrasse 340, 8008 Zurich, Switzerland

**Keywords:** Computed tomography, X-ray, Arthrography, Shoulder, Image enhancement

## Abstract

**Objective:**

To evaluate the image quality of dual energy CT (DECT) of the shoulder after arthrography and of virtual non-contrast (VNC) 3D reformats of the glenoid and to compare glenoid measurements on VNC 3D reformats and on 2D CTs.

**Materials and methods:**

DECT arthrography (80 kV/140 kV) was performed in 42 shoulders of 41 patients with instability using diluted iodinated contrast media (80 mg/ml). VNC images and VNC 3D reformats of the glenoid were calculated using image postprocessing. Dose parameters, CT values of intraarticular iodine and muscle, image contrast (iodine/muscle), and image quality (5-point scale: 1 = worst, 5 = best) were evaluated. Two independent readers assessed glenoid morphology and performed glenoid measurements on 2D and 3D images.

**Results:**

Calculation of VNC images and VNC 3D reformats was successful in 42/42 shoulders (100%). The effective dose was mean 1.95 mSv (± 0.9 mSv). CT values of iodine and muscle were mean 1014.6 HU (± 235.8 HU) and 64.5 HU(± 8.6 HU), respectively, and image contrast was mean 950.2 HU (± 235.5 HU). Quality of cross-sectional images, VNC images, and VNC 3D reformats was rated good (median 4 (4–5), 4 (3–4), 4 (3–5), respectively). Detection of an osseous defect was equal on 2D and 3D images (13/42, *P* > 0.99) with no difference for measurement of the glenoid diameter with mean 28.3 mm (± 2.8 mm) vs. 28.4 mm (± 2.9 mm) (*P* = 0.5), width of the glenoid defect with 3.2 mm (± 2.1 mm) vs. 3.1 mm (± 2.3 mm) (*P* = 0.84), surface area with 638.5 mm^2^ (± 127 mm^2^) vs. 640.8 mm^2^ (± 129.5 mm^2^) (*P* = 0.47), and surface area of the defect with 46.6 mm^2^ (± 44.3 mm^2^) vs. 47.2 mm^2^ (± 48.0 mm^2^) (*P* = 0.73), respectively.

**Conclusion:**

DECT shoulder arthrography is feasible and allows successful iodine removal with generation of VNC images and accurate VNC 3D reformats of the glenoid for assessment of bone loss.

**Supplementary Information:**

The online version contains supplementary material available at 10.1007/s00256-021-03916-3.

## Introduction

The 3D reformats of shoulder CT examinations generated with the volume rendering technique (VRT) are routinely used for assessment of the glenoid morphology and for quantitative measurements and are highly accurate. They are often used during preoperative planning in shoulder instability procedures, especially as the 3D visualization is preferred by the surgeons over the 2D CT reformats [1–7]. The 3D VRT reformats are calculated from single energy CT (SECT) data during clinical routine examinations using commercially available postprocessing software. In a subset of patients with glenoid dysplasia or previous shoulder dislocation, arthrography is performed before the CT scan to evaluate the labrum, cartilage, and the rotator cuff.

However, for SECT scans after arthrography, calculation of 3D VRT reformats is practically useless, because intraarticular iodinated contrast material overlays bone and cannot be successfully separated. In contrast to SECT, dual energy CT (DECT) allows for characterization of tissues (e.g., iodine) according to different attenuation values at different energy levels [8, 9]. DECT has been successfully used for imaging of gout crystal depositions [10] or metal artefact reduction after metal implants [11]. However, for CT arthrography, DECT has only been tested in vitro in cow femoral condyles [12] and porcine joint cadavers [13] and was applied to shoulder patients for evaluation of labral tears [14] and for successful bone-iodine differentiation using material decomposition but without generating virtual non-contrast (VNC) images [15] or glenoid evaluation [16].

We set out to use dual energy CT after arthrography of the shoulder for clinical routine examinations. Our hypothesis was that DECT arthrography provides good image quality and allows for successful calculation of virtual non-contrast images and of accurate VNC 3D VRT reformats of the glenoid for assessment of bone loss.

Therefore, the purpose of this study was to evaluate the image quality of DECT scans of the shoulder after arthrography and of VNC 3D VRT reformats of the glenoid. Furthermore, to compare glenoid measurements on VNC 3D VRT reformats and on 2D CTs.

## Materials and methods

The cantonal ethics committee approved this single center retrospective study which was conducted according to the Declaration of Helsinki.

### Study population

A search of the picture archiving and communication system (PACS) of Balgrist University Hospital was performed to find patients who received a clinical dual energy CT scan of the shoulder after arthrography. Inclusion criteria were males and females aged 18 years or older with shoulder instability. Patients with an incorrect dosage of intraarticular contrast material at arthrography or with metallic screws in the glenoid after surgery were excluded.

### Dual energy CT technique

All patients received a dual energy CT scan of the shoulder at Balgrist University Hospital either on a 128-slice CT scanner (SOMATOM Edge Plus, Siemens Healthineers, Erlangen, Germany; CT 1) or on a 64-slice CT scanner (SOMATOM Definition AS, Siemens Healthineers, Erlangen, Germany; CT 2) within 15 min after arthrography. The scan protocol was adapted from the protocol for the liver VNC application since no shoulder-specific manufacturer settings were available: All scans were performed in sequential technique with a 80 kV scan followed by a second scan with 140 kV of the same coverage in *z*-axis. Both CT scanners operated with automated tube current modulation (CARE Dose4D, reference 240 mAs for 80 kV and 57 mAs for 140 kV), a collimation width of 0.6 mm, a rotation time of 0.5 s, and a pitch of 0.8. The dose settings of the DECT scan were adjusted to the parameters of a single energy scan of the shoulder at 120 kV (reference 150 mAs). The applied total dose was split automatically between the 80 kV and the 140 kV scan by the CT machine.

### Arthrography technique

Injection of diluted iodinated contrast material into the glenohumeral joint at Balgrist University Hospital is routinely performed under conventional fluoroscopy using an anterior approach through the rotator cuff interval [17]. As there is a linear relationship of attenuation and iodine concentration for all tube voltages, the dilution of the iodinated contrast material was adapted in order to not saturate the detector of the CT machine, which occurs at 3071 HU [12]. The total injected volume was 12 ml for all patients. For all injections, a solution of 80 mg iodine per milliliter was used which was achieved by injecting 1 ml of local anesthetics followed by 11 ml of diluted Iopamiro 200 (Iopamidol): 7 ml Iopamiro 200 + 9 ml NaCl 0.9%. With this approach, the intraarticular iodine enhancement stayed below the saturation point of the CT detector for the 80 kV scan, which is a precondition for successful calculation of virtual non-contrast images.

### Image reconstruction and postprocessing

Both the 80 kV and 140 kV scans were reconstructed in the axial image plane (0.75 mm) using a bone (Br 51) and a soft tissue kernel (Qr 40). Furthermore, a blended axial CT arthrogram (CT-A) dataset (0.75 mm) was calculated from the 80 kV and 140 kV scans in bone kernel (Br 51) using a mixing ratio of 0.3:0.7, from which axial (2 mm), sagittal oblique (2 mm), sagittal en face (1 mm), and coronal oblique (2 mm) CT arthrogram images were reconstructed.

**Fig. 1:**
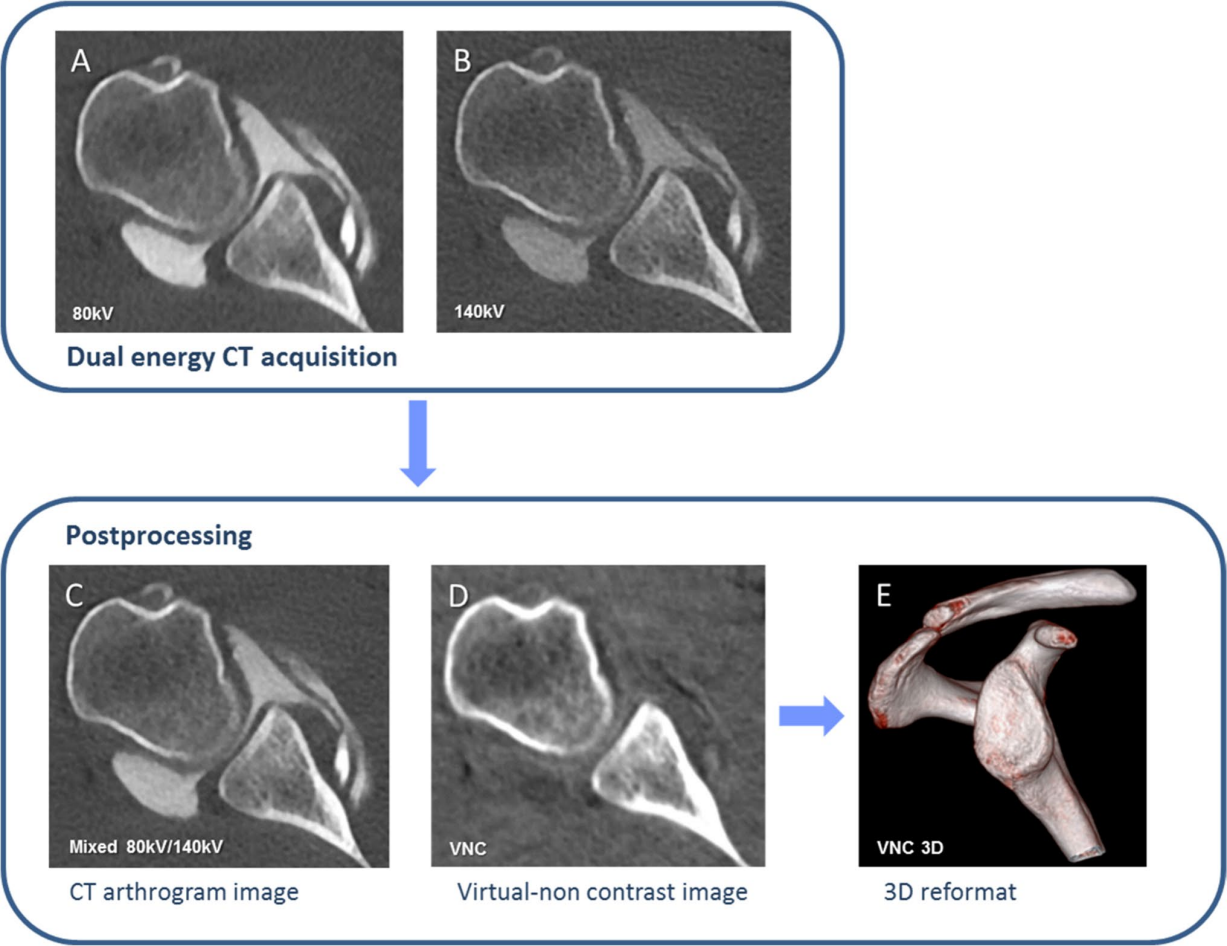
Image acquisition and workflow of dual energy CT after arthrography of the shoulder. The dual energy CT scan acquires 2 datasets, one with 80 kV tube voltage (**A**) and another one with 140 kV (**B**). With image postprocessing, mixed CT arthrogram images (80 kV/140 kV; C) using a mixing ratio of 0.3:0.7 and virtual non-contrast images (**D**) are calculated from (**A**) and (**B**). A VNC 3D osseous reformat of the glenoid (**E**) is calculated from (**D**) using the volume rendering tool

For the calculation of 3D VRT reformats, both the axial 80 kV and 140 kV dataset with a 0.75-mm section thickness in soft tissue kernel (Qr 40) were loaded into the dual energy viewer of syngo.via (VB 30, Siemens Healthineers, Erlangen, Germany). First, virtual non-contrast images with a 0.75-mm section thickness were calculated with the shoulder VNC application which was adapted from the liver VNC application to display higher Hounsfield units. Second, 3D osseous reformats of the glenoid were generated from the VNC images using the volume rendering tool in syngo.via (Fig. 1).

### Image analysis

Images were anonymized and interpreted independently by two musculoskeletal radiologists (C.S. (reader 1) with 7 and M.M. (reader 2) with 6 years of experience) on a PACS workstation. Both readers were blinded to each other and were blinded to clinical information and imaging results.

#### Quantitative image analysis

Scan length and CT dose parameters were extracted from the dose report: tube current–time product (mAs), volume CT dose index (CTDIvol), and dose length product (DLP). The DLP was multiplied with a standard conversion factor *k* for the adult chest of 0.014 mSv/mGy*cm to estimate the effective dose [18].

CT values (HU) of the intraarticular iodinated contrast material were measured by reader 1 on reconstructed axial 0.75-mm images of the 80 kV scan in soft tissue kernel and on blended axial CT arthrogram images (0.75 mm) using regions of interest (ROI) of equal size (20 mm^2^). The copy and paste function of the PACS was used, allowing to position the ROIs at the identical location in both datasets. Furthermore, CT values (HU) of the deltoid muscle were also measured on the blended axial CT-A images using ROIs of the same size. Image contrast of blended CT-A images was calculated as the difference of CT values of intraarticular iodinated contrast material and deltoid muscle.

#### Qualitative image analysis

Reader 1 rated the following parameters on a 5-point Likert scale: overall image quality of the blended CT-A images (1 = poor, 2 = fair, 3 = moderate, 4 = good, 5 = excellent), image quality of virtual non-contrast images (1 = poor, 2 = fair, 3 = moderate, 4 = good, 5 = excellent), and image quality of virtual non-contrast 3D VRT reformats of the glenoid (1 = poor, 2 = fair, 3 = moderate, 4 = good, 5 = excellent). Ratings are defined in Supplementary Table 1.

In a sub-analysis, the image quality was evaluated separately for the different CT systems.

Furthermore, CT arthrogram images of patients with surgical correlation were assessed for rotator cuff and labrum tears and for cartilage defects.

#### Glenoid measurements

Glenoid measurements were performed on sagittal en face 2D arthrogram images (1 mm) and on en face VNC 3D VRT reformats of the glenoid by both readers. The best fit-circle method was applied for all measurements, which assumes that the inferior shape of the glenoid fits a perfect circle [2, 3, 19, 20]. The glenoid diameter and the glenoid surface area (Pico method) were measured on both 2D and 3D images. Both image sets were evaluated for the presence of an osseous defect. Defect size was measured as width of the glenoid defect in millimeter (mm) [19, 21] and as surface area of the defect in square millimeter (mm^2^) for both 2D and 3D images [2, 3]. Reader 1 first interpreted 2D images and then 3D images, whereas reader 2 evaluated the images in the opposite order. For both readers, the interval between readouts was 2 months.

### Statistical analysis

We used general descriptive statistics and reported ordinal data as median with 25th percentile (Q1) and 75th percentile (Q3) and continuous data as mean with standard deviation (SD). To test for normal distribution, the Shapiro–Wilk test was used.

Prevalence of an osseous defect was evaluated on reconstructed 2D CT-A images and on VNC 3D VRT reformats, and the McNemar test was used for comparison. We used Bland–Altman plots [22] and the paired t-test to compare glenoid measurements on 2D and 3D images. Sub-analysis was performed to test for differences in measurements between the two CT systems. Interreader agreement was measured with intraclass correlation coefficients (ICC): ICC values > 0.75 were interpreted as good and > 0.9 as excellent agreement [23].

SPSS (Version 25, IBM Corporation, Armonk, NY) was used for statistical analysis with a level of significance of < 0.05 for any value of *P*.

## Results

### Study participants

The PACS query revealed 52 patients with shoulder instability who received a clinical dual energy CT of the shoulder after arthrography. Six patients declined informed consent and were therefore excluded. Four patients were excluded because of metal implants and one patient because of incorrect dosage of intraarticular contrast material at arthrography. The resulting study group included 41 patients (31 male, 10 female; mean age 33.4 years ± 13.9 years [standard deviation]) and 42 shoulders (1 male patient received DECT after arthrography of both shoulders).

### CT parameters, effective dose, and quantitative analysis

CT parameters of the DECT of the shoulder after arthrography are listed in Table 1. The estimated effective dose of the dual energy CT scans after arthrography was mean 1.95 mSv (± 0.9 mSv).

**Table 1:** Scan length and CT dose parameters of patient scans

	Dual energy CT shoulder after arthrography
Tube current	80 kV, 140 kV
Tube current–time product	80 kV: 302 mAs (± 168 mAs) 140 kV: 57 mAs (± 19 mAs)
CTDIvol	11.7 mGy (± 5.2 mGy)
DLP	139.3 mGy*cm (± 66.4 mGy*cm)
Scan length	118 mm (± 11 mm)
Effective dose†	1.95 mSv (± 0.9 mSv)

CT parameters were automatically adapted to patient size

^†^Note. Effective dose (mSv) was estimated by multiplying the DLP with a standard conversion factor k for the adult thorax of 0.014 mSv/mGy*cm

Values are displayed as mean with standard deviation in parentheses

Abbreviations: *CTDIvol* volume CT dose index, *DLP* dose length product, *kV* kilo volt, *mAs* milliampere seconds, *mGy* milligray, *mSv* millisievert

Calculation of the blended CT arthrogram images, of virtual non-contrast images and of VNC 3D VRT reformats of the glenoid, was successful for all shoulders (42/42, 100%).

CT values of the intraarticular iodinated contrast material were mean 1542.4 HU (± 369 HU) for the 80 kV scan in soft tissue kernel and 1014.6 HU (± 235.8 HU) for the blended CT arthrogram images. The mean CT value of the deltoid muscle measured on CT-A images was 64.5 HU (± 8.6 HU). The image contrast of CT-A images was mean 950.2 HU (± 235.5 HU) between intraarticular iodinated contrast material and deltoid muscle.

### Qualitative image analysis

**Fig. 2:**
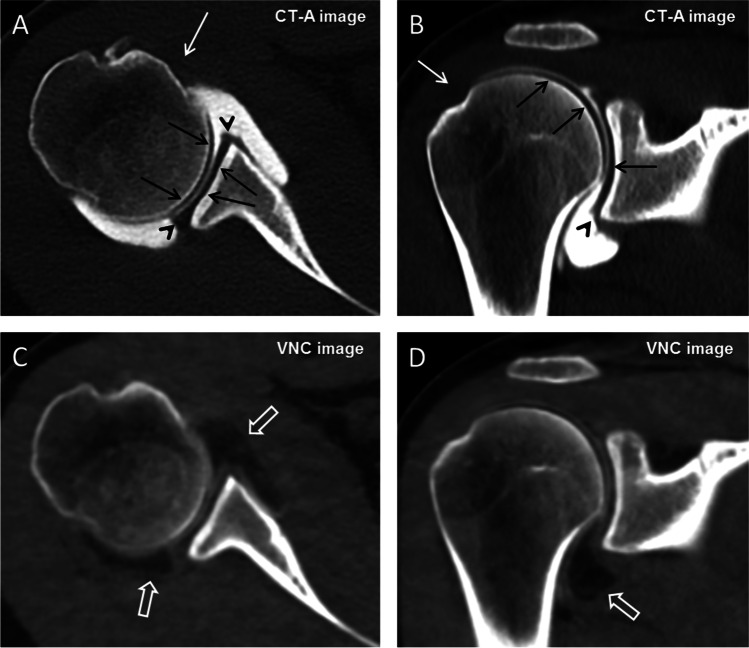
**A** 20-year-old female with anterior glenohumeral instability. Dual energy CT after arthrography of the right shoulder with reformatted axial (**A**) and coronal oblique (**B**) CT arthrogram (CT-A) images clearly show the articular cartilage (black arrows), labrum (black arrow heads), and tendons of the rotator cuff (white arrows). The cortical and trabecular structure of the humeral head and of the glenoid is clearly visible. Reformatted axial (**C**) and coronal oblique (**D**) images of the virtual non-contrast (VNC) dataset show full subtraction of the intraarticular iodinated contrast material (open arrows). Note substance loss and fraying of the inferior labrum in (**B)** because of Bankart lesion. Image contrast between iodine and soft tissue was 1170 Hounsfield units

**Fig. 3:**
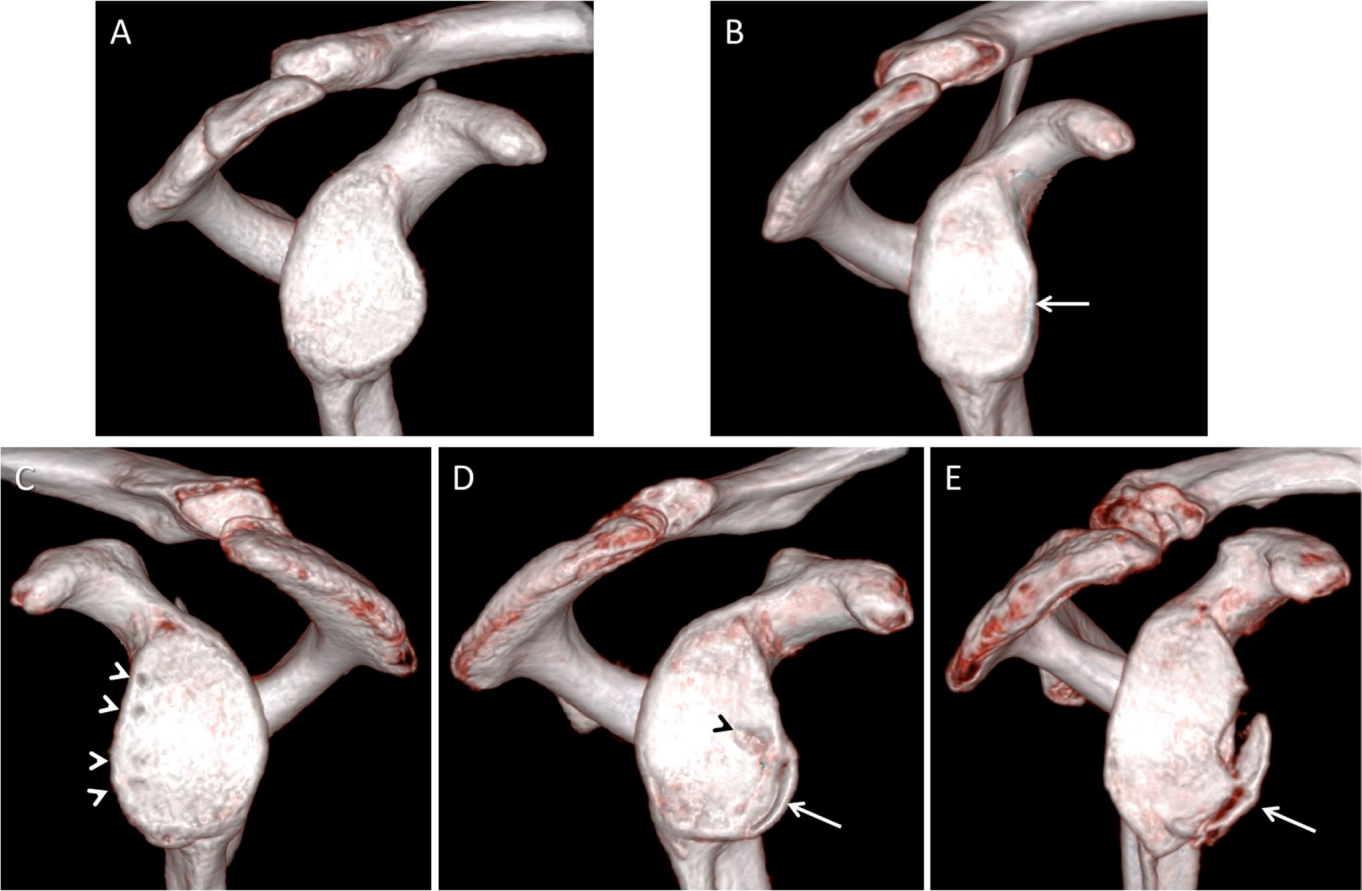
Examples of VNC 3D VRT reformats of the glenoid. **A** A 25-year-old male with normal shaped glenoid without bone loss. **B** A 20-year-old female with anterior glenohumeral instability. The anteroinferior glenoid appears straight suggesting a compression fracture (arrow). **C** A 25-year-old male with evidence of anterior labrum repair with visible drill holes (arrow heads). **D** A 22-year-old male with partial fibrous consolidation of a small anteroinferior osseous Bankart fragment (arrow) and a small osseous cyst (arrow head). **E** A 59-year-old male with recurrent shoulder instability and a large displaced osseous Bankart fragment anterior (arrow)

CT arthrogram images, VNC images, and VNC 3D VRT reformats were of good quality: The overall image quality of the CT-A images was median 4 (4–5), the image quality of virtual non-contrast images was median 4 (3–4), and the image quality of virtual non-contrast 3D VRT reformats of the glenoid was median 4 (3–5) (Figs. 2 and 3).

For CT 1 and CT 2, the image quality of CT arthrogram images, VNC images, and VNC 3D VRT reformats were median 4 (4–5) and 5 (4–5), 4 (3–4) and 4.5 (4–5), and 4 (3–4.25) and 5 (3.25–5), respectively. However, the number of patients scanned on CT 1 was more than 4 times higher than on CT 2 (34 vs. 8 patients).

Sub-analysis of CT arthrogram images of patients with shoulder surgery (*n* = 8) revealed labrum tears in 5 patients, rotator cuff tears in 1 patient, and cartilage defects in 3 patients, all of which were confirmed surgically.

### Glenoid measurements

Glenoid measurements were successful on all sagittal en face 2D CT arthrogram images (42/42, 100%) and on all en face VNC 3D VRT reformats of the glenoid (42/42, 100%). No difference was observed for the glenoid diameter with mean 28.3 mm (± 2.8 mm) measured on 2D images compared to mean 28.4 mm (± 2.9 mm) measured on VNC 3D VRT reformats (*P* = 0.5). The glenoid surface area was also not different with mean 638.5 mm^2^ (± 127 mm^2^) on 2D images vs. mean 640.8 mm^2^ (± 129.5 mm^2^) on VNC 3D images (*P* = 0.47).

**Fig. 4:**
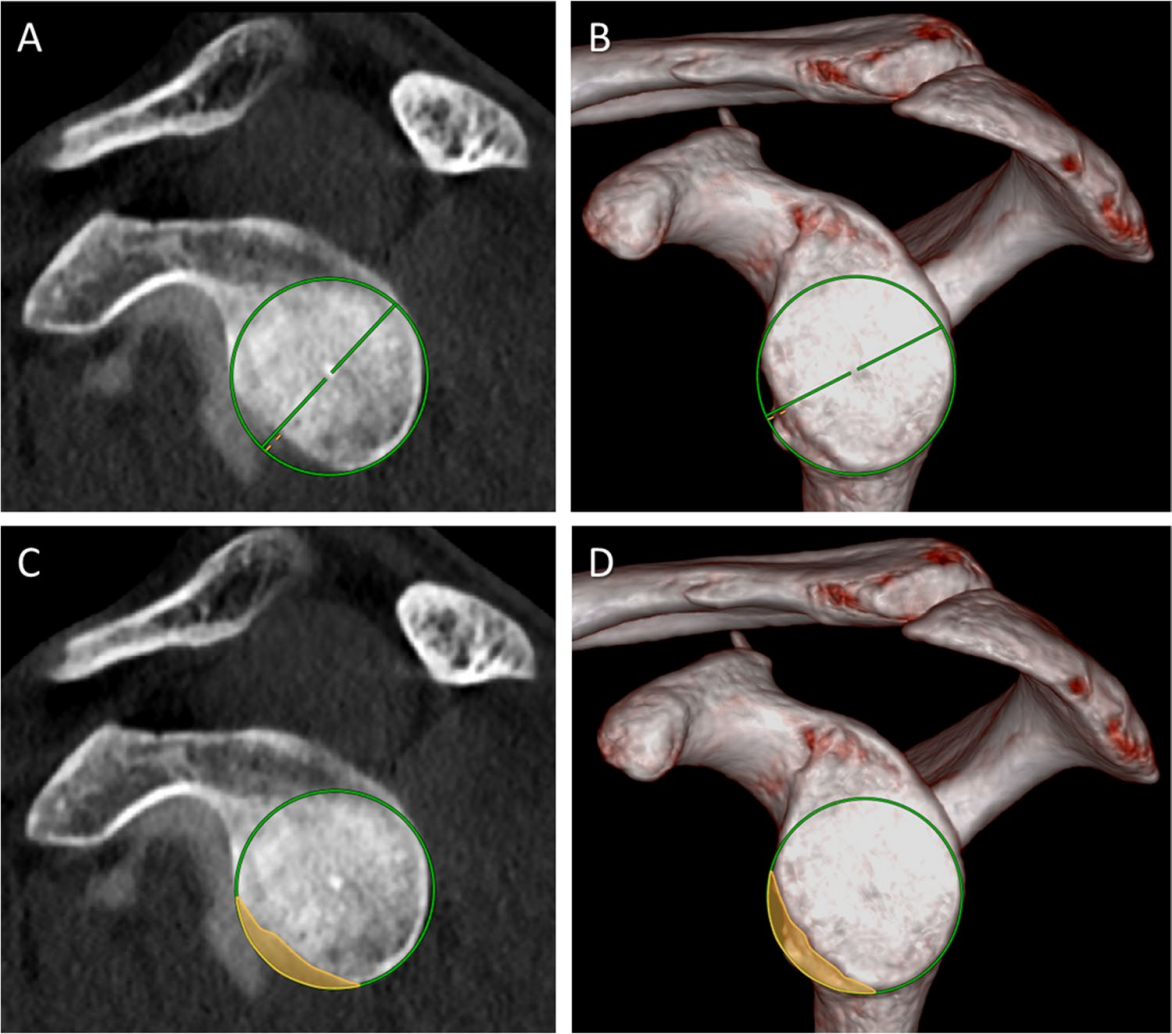
A 28-year-old male with bony Bankart lesion of the anteroinferior glenoid. Dual energy CT after arthrography of the right shoulder with reformatted sagittal en face 2D arthrogram images (**A** and **C**) and en face 3D VNC VRT reformats of the glenoid (**B** and **D**). Measurement of the glenoid diameter (green lines) using the best fit circle method was comparable with 30.9 mm on the 2D image (**A**) and 31.4 mm on the 3D image (**B**). The width of the glenoid defect was measured identical with 3.1 mm (yellow lines in (**A**) and (**B**)). The glenoid surface area (green circles) was also measured similar on the 2D image (**C**) and 3D image (**D**) with 751 mm2 and 775 mm2, respectively. Similarly, the surface area of the osseous glenoid defect (yellow areas) was measured with 49 mm2 on image (**C**) and 44 mm2 on image (**D**)

**Fig. 5:**
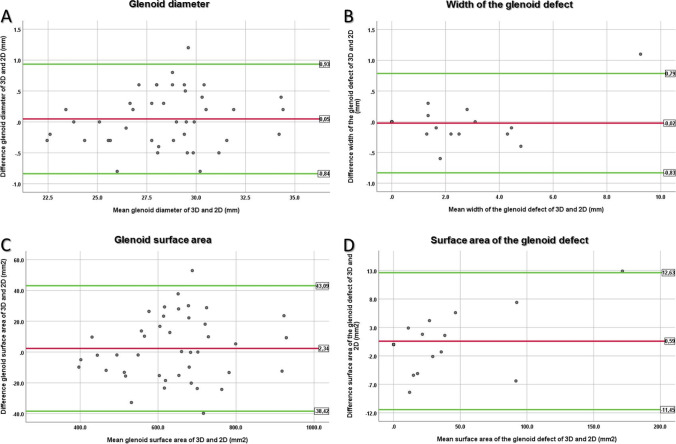
Bland–Altman plots for the glenoid diameter (**A**), width of the glenoid defect (**B**), glenoid surface area (**C**), and surface area of the glenoid defect (**D**). The upper limit, mean value, and lower limit are displayed in the boxes on the right of each plot. Note: Data in (**A**) and (**B**) are displayed in millimeters (mm) and in (**C**) and (**D**) in square millimeters (mm^2^)

Detection of an osseous defect was equal on sagittal en face 2D CT-A images (13/42, 31%; 95% confidence interval [CI]: 18.6%, 45.8%) and on en face VNC 3D VRT reformats (13/42, 31%; 95% CI: 18.6%, 45.8%) (*P* > 0.99) with no discrepant cases. There was no difference for the width of the osseous glenoid defect with mean 3.2 mm (± 2.1 mm) measured on 2D images compared to 3.1 mm (± 2.3 mm) measured on VNC 3D images (*P* = 0.84), nor was the surface area of the osseous glenoid defect measured different with mean 46.6 mm^2^ (± 44.3 mm^2^) and 47.2 mm^2^ (± 48.0 mm^2^) (*P* = 0.73), respectively (Fig. 4). Figure 5 shows the corresponding Bland–Altman plots. The range between the lower and upper limit was as follows: 1.8 mm for the glenoid diameter, 81.5 mm^2^ for the glenoid surface area, 1.6 mm for the width of the osseous glenoid defect, and 24.1 mm^2^ for the surface area of the osseous glenoid defect. Sub-analysis also revealed no difference for each category of glenoid measurements performed on 2D and 3D images for examinations on CT 1 (*n* = 34; *P* = 0.76–0.99) or on CT 2 (*n* = 8; *P* = 0.36–0.40).

Agreement between readers for all glenoid measurements was excellent on 2D images (ICC 0.98) and excellent on VNC 3D VRT reformats (ICC 0.98).

## Discussion

We showed that DECT shoulder arthrography is feasible for clinical routine examinations and allows successful iodine removal using material decomposition with generation of virtual non-contrast (VNC) images and accurate 3D VRT reformats of the glenoid for assessment of bone loss.

With a concentration of 80 mg iodine per ml contrast solution injected into the joint we were able to acquire DECT arthrogram images in diagnostic quality and successfully calculate both cross-sectional VNC images as well as VNC 3D VRT reformats for all clinical examinations. Subhas et al. showed diagnostic image quality for CT scans of human knee cadavers performed in single energy technique with 80 kV after arthrography using 60 mg iodine per ml solution. Furthermore, they demonstrated that the saturation point of the CT detector was reached at 93 mg iodine per ml solution for both the 80 kV SECT and the 80 kV scan of the 80 kV/140 kV DECT scan in a phantom [12].

An et al. determined in a 2014 phantom study the optimal contrast dose at 60 mg iodine/ml and the energy level at 72 keV for DECT arthrography of the shoulder using virtual monochromatic spectral imaging. The optimized protocol was then applied to 23 patients, which resulted in 36% reduction of image noise and in 45% reduction of beam-hardening artifacts compared to the standard protocol [15]. The authors only evaluated material decomposition; however, no VNC images or 3D VNC reformats were calculated.

In our study, evaluating 42 shoulders in 41 patients, we observed excellent image contrast with a mean difference of 950 HU between the intraarticular iodinated contrast material and the soft tissues on CT-A images, which allows to confidently assess the labrum, the rotator cuff, and the articular cartilage for pathology. In all of our patients with subsequent shoulder surgery (*n* = 8), the labrum, rotator cuff, and cartilage lesions identified on CT-A images were surgically confirmed. In their study with 47 shoulder patients, Foti et al. demonstrated that DECT arthrography is equal to magnetic resonance arthrography in detecting labral tears [14]. Further, our results were in accordance with the results of An et al., who found an image contrast above 800 HU between the intraarticular iodine contrast agent and soft tissues for the optimized virtual monochromatic spectral imaging DECT protocol with 60 mg iodine/ml [15]. Therefore, we believe that 80 mg iodine/ml solution is appropriate in DECT shoulder arthrography for both accurate assessment of the internal structures and successful iodine removal. The virtual monochromatic imaging technique, however, only decreases the attenuation of iodinated contrast material at high keV and is inferior to the VNC technique in generating virtual non-contrast images, which was shown by the results of Sandhu et al. [16]. We used the virtual unenhanced technique to calculate virtual non-contrast images from the 80 kV and 140 kV DECT dataset using the customized liver VNC application in syngo.via, which allowed for subtraction of iodinated intraarticular contrast agent.

Chai et al. also used the virtual unenhanced technique in an in vitro study with porcine joints and showed successful calculation of virtual non-contrast images and VNC 3D osseous reformats from DECT scans (80 kV/140 kV) at 75 mg iodine/ml. In their study, subtraction and image calculation failed for all higher contrast agent dosages, because the demonstrable upper HU limit of the CT detector was reached and consequently the subtraction algorithm of the VNC application was unable to recognize the material as iodine [12, 13]. Our study results were in accordance with successful calculation of VNC images and VNC 3D VRT reformats for all study participants using 80 mg iodine/ml.

Our results showed that detection of an osseous defect of the glenoid was accurate on VNC 3D VRT reformats, with no discrepant cases compared to 2D CT. In a study with 7 shoulder cadavers, Rerko et al. also demonstrated a high correlation between true anteroinferior glenoid bone loss vs. predicted bone loss on 3D CT (*r* = 0.875) and on 2D CT (*r* = 0.831), respectively, with comparable prediction errors for 3D CT and 2D CT. They recommend to use 3D CT as the most accurate, reliable, and reproducible imaging modality to estimate anteroinferior glenoid bone loss [4].

Sugaya et al. evaluated glenoid morphology in 100 shoulders with recurrent anterior glenohumeral instability and found that in shoulders with abnormal morphology discovered on 3D CT, appearance during arthroscopy was comparable [5].

Margarelli et al. discovered 97% agreement between 2D CT and 3D VRT reformats in detecting an osseous defect of the glenoid in 100 patients with unilateral anterior glenohumeral instability. Furthermore, they showed a mean difference of 0.62% between the 2 methods for measurement of the percentage of glenoid bone loss using the Pico method [7]. Our study results were in accordance as we did not find a statistically significant difference between 2D CT and VNC 3D VRT reformats for measurement of the glenoid diameter (*P* = 0.5), the glenoid surface area (*P* = 0.47), the width of the glenoid defect (*P* = 0.84), or the surface area of the glenoid defect (*P* = 0.73). Therefore, we conclude that the subtraction algorithm of the VNC application works properly and 3D VRT reformats are accurate.

With a CTDIvol of mean 11.7 mGy and an effective dose of mean 1.95 mSv, the dual energy CT scans of our study were below the radiation dose reported in the literature. An et al. reported a CTDIvol of 17.8 mGy for DECT arthrography of the shoulder in 23 patients using virtual monochromatic spectral imaging [15]. Biswas et al. reported a CTDIvol of mean 19.5 mGy and an effective dose of mean 2.06 mSv for non-contrast CT scans of the shoulder with 120 kV in 20 patients [24].

A limitation of our study was that patients were scanned on 2 different CT scanners. However, acquisition parameters were adjusted to equal dose, and the same dual energy protocols were used, which resulted in comparable image quality and no difference in glenoid measurements according to a sub-analysis for examinations performed on CT 1 and 2. Furthermore, the dual energy CT protocol incorporated a 70-s delay between the 80 kV and the 140 kV scan since it was derived from the liver VNC protocol. In order for the VNC application to work, deactivation of the delay was not possible. However, we did not experience significant patient motion during the two scans which could possibly negatively affect image quality.

In summary, dual energy CT arthrography of the shoulder for clinical routine examinations is feasible and showed good image quality at a dose comparable to single energy shoulder CT. With image postprocessing, diagnostic virtual non-contrast 3D VRT reformats of the glenoid were successfully calculated, which allow for 3D assessment of glenoid morphology and bone loss and accurate glenoid measurements.

## Supplementary Information

Below is the link to the electronic supplementary material.

Supplementary file1 (DOCX 85 KB)

## Abbreviations

CTDIvol: Volume CT dose index
DECT: Dual energy CT
DLP: Dose length product
HU: Hounsfield unit
kV: Kilo volt
mAs: Milliampere seconds
mGy: Milligray
mSv: Millisievert
ROI: Region of interest
SECT: Single energy CT
VNC: Virtual non-contrast
VRT: Volume rendering technique
