# Supplementary figures and images for: Dual energy CT arthrography in shoulder instability: successful iodine removal with virtual non-contrast images and accurate 3D reformats of the glenoid for assessment of bone loss
Source: Skeletal Radiol. 2021 Oct 3;51(5):1027–36. doi: 10.1007/s00256-021-03916-3 (PMC8930895; doi:10.1007/s00256-021-03916-3)

**Definition of ratings on 5-point Likert scale**


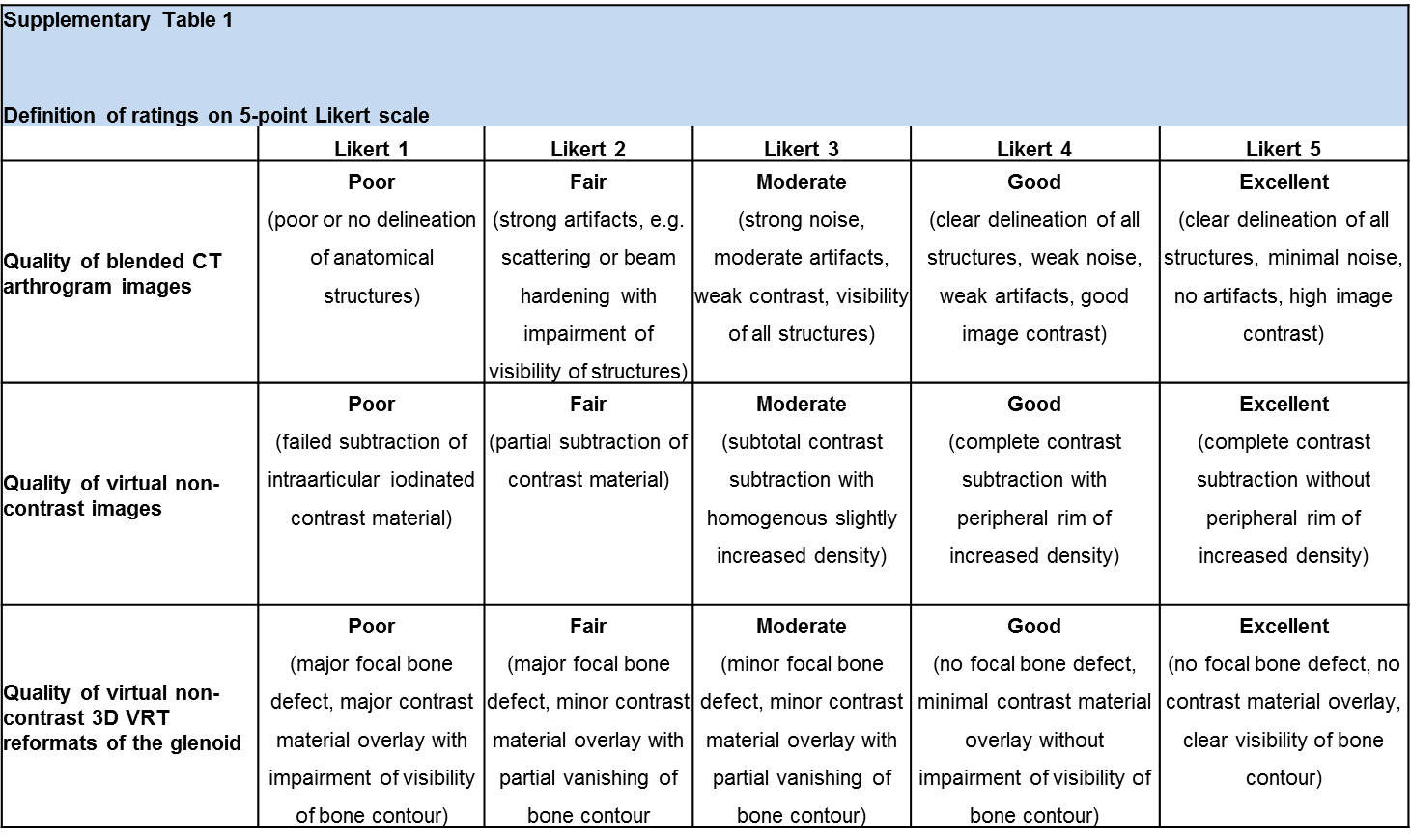

Supplement: Supplementary file 1 — Supplementary file1 (DOCX 85 KB) [file 256_2021_3916_MOESM1_ESM.docx]
